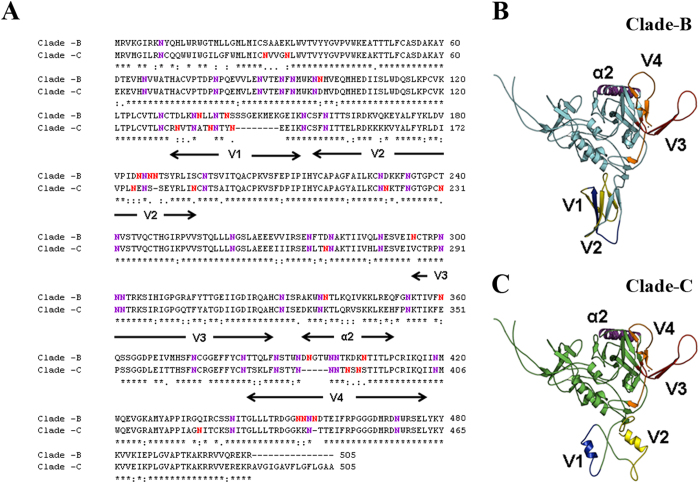# HIV Subtypes B and C gp120 and Methamphetamine Interaction: Dopaminergic System Implicates Differential Neuronal Toxicity

**DOI:** 10.1038/srep11130

**Published:** 2015-06-09

**Authors:** Thangavel Samikkannu, Kurapati V. K. Rao, Abdul Ajees Abdul Salam, Venkata S. R. Atluri, Elena M. Kaftanovskaya, Marisela Agudelo, Suray Perez, Changwon Yoo, Andrea D. Raymond, Hong Ding, Madhavan P. N. Nair

**Affiliations:** 1 Department of Immunology, Institute of NeuroImmune Pharmacology, Herbert Wertheim College of Medicine, Florida International University, Miami, Florida, USA; 2 Department of Atomic and Molecular Physics, Manipal University, Manipal 576104, Karnataka, India; 3 Department of Human and Molecular Genetics, Herbert Wertheim College of Medicine, Florida International University, Miami, Florida, USA; 4 Department of Epidemiology and Biostatistics, School of Public Health, Florida International University, Miami, Florida 33199.

## Abstract

HIV subtypes or clades differentially induce HIV-associated neurocognitive disorders (HAND) and substance abuse is known to accelerate HIV disease progression. The HIV-1 envelope protein gp120 plays a major role in binding and budding in the central nervous system (CNS) and impacts dopaminergic functions. However, the mechanisms utilized by HIV-1 clades to exert differential effects and the methamphetamine (METH)-associated dopaminergic dysfunction are poorly understood. We hypothesized that clade B and C gp120 structural sequences, modeling based analysis, dopaminergic effect, and METH potentiate neuronal toxicity in astrocytes. We evaluated the effect of clade B and C gp120 and/or METH on the DRD-2, DAT, CaMKs and CREBP transcription. Both the structural sequence and modeling studies demonstrated that clade B gp120 in V1-V4, α -2 and N-glycosylated sites are distinct from clade C gp120. The distinct structure and sequence variation of clade B gp120 differentially impact DRD-2, DAT, CaMK II and CaMK IV mRNA, protein and intracellular expression compared to clade C gp120. However, CREB transcription is upregulated by both clade B and C gp120, and METH co-treatment potentiated these effects. In conclusion, distinct structural sequences of HIV-1 clade B and C gp120 differentially regulate the dopaminergic pathway and METH potentiates neurotoxicity.

HIV-1 infection causes immune dysfunction and is a risk factor in the neuropathogenesis of brain disease^1^. HIV-infected brain cells secrete inflammatory cytokines, chemokines and neurotoxic factors that alter amino acid metabolism and neurotransmitter systems, including dopamine, acetylcholine and serotonin. However, HIV infection has a significant effect on dopamine^2^,^3^,^4^,^5^.

Clinical observations suggest that patients with HIV-associated neurocognitive disorders (HAND) may have dopamine deficits associated with cognitive dysfunctions^6^,^7^. HIV infection alters intracellular Ca^2+^, affecting dopamine levels, dopamine receptors (DRD) and the dopamine transporter (DAT)^8^,^9^. In addition, calcium influx exerts its effects on the ubiquitous Ca^2+^ sensor, including the calcium/calmodulin-dependent protein kinases CaMK II and CaMK IV^10^,^11^, which affect the cyclic response element binding protein (CREBP)^12^,^13^. Collectively, dopaminergic systems may be vulnerable to the effects of HIV infection in the brain.

The HIV-1 envelope protein gp120 is required for viral entry and causes neurotoxicity in the central nervous system (CNS)^14^,^15^. Previous studies demonstrated that the HIV-1 gp120 and Tat proteins induce the over-stimulation of intracellular Ca^2+^^16^,^17^, which could affect the dopaminergic system and dysregulate CaMKs and CREB transcription in the CNS^18^,^19^. Illicit drug abuse is a risk factor for HIV infection and AIDS progression. Studies demonstrated that methamphetamine (METH) users^20^,^21^ and HIV-infected METH users have impaired immune function and synergistically potentiated neurotoxicity^22^. We previously reported that METH accelerates HIV infection and HIV-1gp120- and Tat-induced immune and neuronal toxicity^23^,^24^. Recent studies demonstrated that CaMKs and CREB transcription is involved in neurocognition and behavioral disorders associated with polydrug abuse, including METH abuse^25^,^26^.

HIV-1 displays genetic variation and can be classified into approximately 11 sub-types/clades^27^, and the predominant clades (i.e., clades B and C) are found in over 86% of patients globally^28^. The genomic sequence of the HIV-1 clade B and C gp120 suggests that differentiation of the V3 and C3 regions^29^,^30^,^31^ leads to differentially expressed AIDS dementia complex (ADC)^32^. However, the precise mechanism by which clade B and C gp120 exert their effects on the CNS remains unknown. Despite mounting evidence that METH abuse potentiates HIV infection, mechanistic studies addressing the combined effects of METH and HIV infection on the dopaminergic system are lacking in patients with HIV-induced neuropathogenesis. We aim to elucidate the effect of HIV-1 clade B and C gp120 on the dopaminergic system and the mechanisms by which METH potentiates neuronal impairments.

## Results

### HIV-1 clade B and C gp120 inhibit DRD-2 gene expression

**Figure 1:**
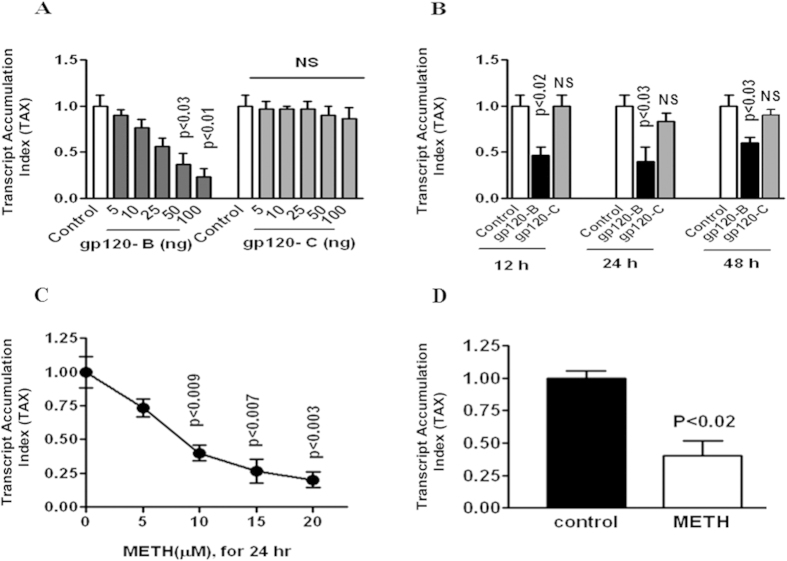
The effect of HIV-1 clade B and C gp120 proteins and METH impact dopaminergic dysfunction in DRD-2 and DAT gene expression. Astrocytes (1 × 10^6^ cells/ ml) were cultured for 24 h without treatment (control) or with HIV-1 clade B gp120 or HIV-1 clade C gp120 treatment (0–100 ng/ml) (**A**) for dose response studies. For kinetic studies, the cells were cultured with 50 ng/ml of clade B and C gp120 protein for 12, 24, and 48 hr (**B**). Cells were cultured for 24 hr with 0- 20 μM METH to assess DRD-2 expression (Fig. 1C) or 10 μM METH to assess DAT expression (Fig. 1D). RNA was extracted, reverse transcribed, and subjected to quantitative real-time PCR using specific primers for DRD-2, DAT and the housekeeping gene β-actin. Data are expressed as the mean ± SE of the TAI values from three independent experiments.

The data presented in Fig. 1A,B show the dose- (0–100 ng/ ml) and time-dependent (50 ng/ml) for 12, 24 and 48 hrs effects of clade B and C gp120 on DRD-2 gene expression in astrocytes, as assessed using quantitative real-time PCR. Astrocytes treated with clade B gp120 significantly down regulated DRD-2 gene expression at 50 ng (p < 0.03) and 100 ng (p < 0.01) compared to gp120 from clade C. The F value for the ANOVA with post-hoc test is 10.112 in calde B. Further, significant downregulation of DRD-2 gene expression by clade B gp120 was observed at 12 (p < 0.02), 24 (p < 0.03) and 48 hr (p < 0.03) compared to clade C gp120 and the untreated control analyzed by one –way ANOVA statistical method.

### Effect of METH on DRD-2 and DAT gene expression

The effect of METH on DRD-2 and DAT expression in astrocytes was examined. The results presented in Fig. 1C,D show the effect of 24-hr incubation with METH on DRD-2 (5 μM- 20 μM METH) and DAT (10 μM METH) expression, as assessed using qRT-PCR. METH treatment significantly down-regulated DRD-2 gene expression in astrocytes at 10 μM (p < 0.009), 15 μM (p < 0.007) and 20 μM (p < 0.003) in a concentration dependent manner. The F value for the ANOVA is 12.01198. The data presented in Fig. 1D show that 10 μM METH significantly downregulated DAT gene expression (p < 0.02) in astrocytes compared to the untreated control analyzed by t-test independent sample. These results indicate that METH induces a dose-dependent suppression of DRD-2 and further down regulated DAT gene expression in astrocytes by 24 hr.

### gp120 induced inhibition of DRD-2 and DAT gene expression potentiated by METH

**Figure 2:**
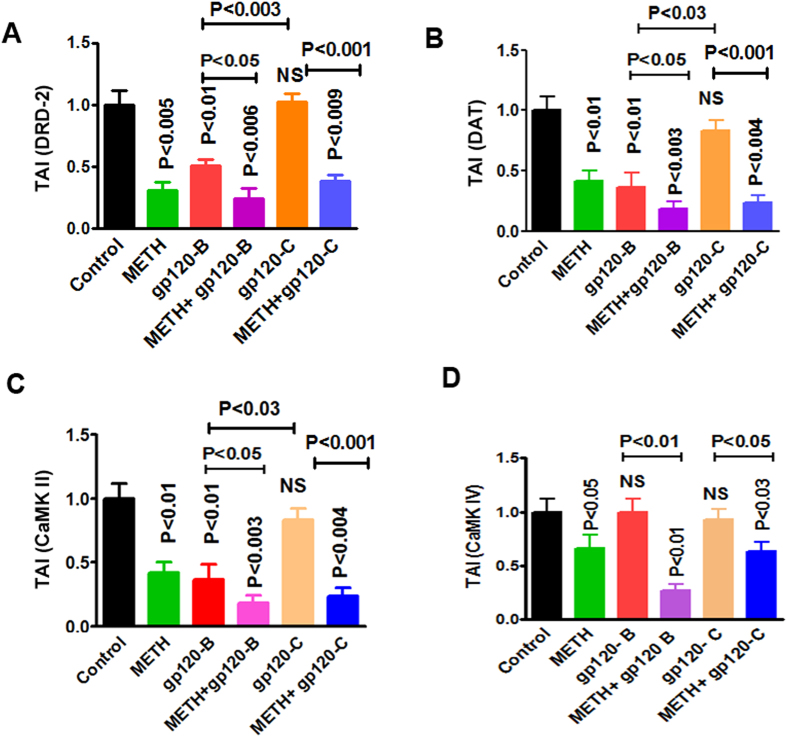
HIV-1 clade B and C gp120 and METH co-morbidity impact dopaminergic dysfunction and CaMKs gene expression. METH has synergistic effects with HIV-1 clade B and C gp120 on the downregulation of DRD-2 (**A**), DAT (**B**), CaMK II (**C**) and CaMK IV (**D**) gene expression. Astrocytes (1 × 10^6^ cells/ ml) were cultured for 24 h without treatment (control) or with METH (10 μM), clade B gp120 (50 ng/ ml), or clade C gp120 (50 ng/ ml) alone or METH in combination with clade B or C gp120. RNA was extracted, reverse transcribed, and subjected to quantitative real-time PCR using primers specific for DRD-2 (**A**), DAT (**B**), CaMK II (**C**), CaMK IV (**D**) and the housekeeping gene β-actin. Data are expressed as the mean ± SE of the TAI values from three independent experiments.

Substance abuse primarily affects neurotransmitters and essential amino acids in the CNS, leading to dopaminergic dysfunction and neuronal disorders, such as depression and cognitive and motor dysfunction. However, METH users are more vulnerable to HIV infection and subsequently present with accelerated dopaminergic dysfunctions^33^,^34^. Therefore, the effects of METH at 10 μM, clade B gp120 (50 ng), and clade C gp120 (50 ng) alone or METH in combination with clade B or C gp120 on the expression of DRD-2 and DAT were examined. The data presented in Fig. 2A demonstrate that METH (p < 0.005), clade B gp120 (p < 0.01), METH with clade B gp120 (p < 0.006), and METH with clade C gp120 (p < 0.009) significantly decreased DRD-2, compared to either the untreated control or treatment with clade C gp120 alone. Furthermore, the effect of clade B gp120 combined with METH as well as clade B gp120 with METH showed significant decrease in DRD-2 compared to either clade B gp120 (p < 0.05) or clade C gp120 (p < 0.001) alone (Fig. 2A). The data presented in Fig. 2B demonstrate that METH (p < 0.01), clade B gp120 (p < 0.01), METH combined with clade B gp120 (p < 0.003), and METH combined with clade C gp120 (p < 0.004) significantly down regulated DAT gene expression compared to either the untreated control or treatment with clade C gp120 alone. In addition, the effect of METH combined with clade B gp120 and METH with clade C gp120 showed significant decrease in DAT expression compared to either clade B gp120 (p < 0.05) or clade C gp120 (p < 0.001) alone (Fig. 2B). These results suggest that clade B and C gp120 differentially regulate DRD-2 and DAT gene expression and these effects are potentiated when METH is combined with clade B gp120 or clade C gp120.

### HIV-1 clade B and C gp120 differentially impact CaMKs II and IV in the presence of METH

Dopamine receptors and transporters play a major role in the regulation of CaMKs signaling pathways. The CaMKs signaling cascade has been implicated in the regulation of dopaminergic neurotransmission after chronic administration of METH^35^. In addition, these pathways play a major role in dopaminergic dysfunctions that predispose patients to dementia and neurocognitive disorders. In this study, we examined the effect of clade B gp120 and clade C gp120 alone or in combination with METH on CaMKs (i.e., CaMK II and IV) gene expression using qRT-PCR. The results in Fig. 2C demonstrate that METH (p < 0.01) and clade B gp120 (p < 0.01) alone significantly down-regulated CaMK II compared to the untreated control or treatment with clade C gp120 alone. METH combined with clade B gp120 (p < 0.003) and METH combined with clade C gp120 (p < 0.004) significantly decreased both CaMK II and CaMK IV compared to the untreated control. Furthermore, the combined effects of METH with clade B gp120 (p < 0.05) and METH with clade C gp120 (p < 0.001) showed significant decrease in CaMK II expression compared to either clade B or C gp120.

The data presented in Fig. 2D demonstrate that CaMK IV gene expression was not affected by clade B and C gp120. However, CaMK IV was down-regulated by METH. Interestingly, the combination of clade B gp120 or clade C gp120 with METH significantly down regulates CaMK IV expression compared to the untreated control (p < 0.01 and p < 0.03) respectively. However, the combined effects of METH with clade B gp120 (p < 0.01) and METH with clade C gp120 (p < 0.05) significantly decreased in CaMK IV expression compared to either clade B or clade C gp120. Further, combined treatment with clade B gp120 and METH has higher suppressive effects on CaMK IV expression compared to METH combined treatment with clade C gp120, suggesting that clade B and clade C gp120 differentially affected CaMK II and CaMK IV (Fig. 2C,D). Statistical analysis was performed using two-way ANOVA method.

### METH and clade B and C gp120 effect in DRD-2 proteins

**Figure 3:**
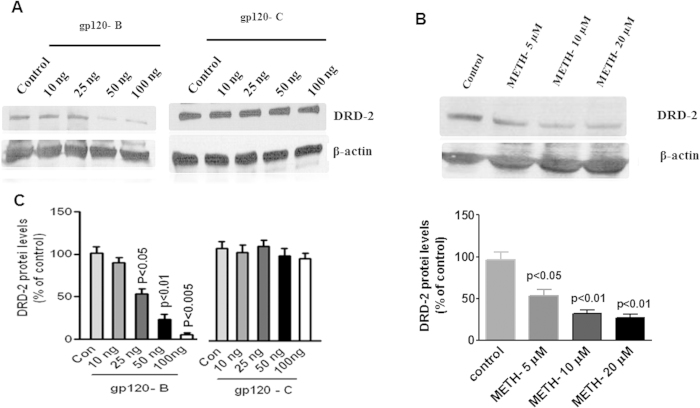
The kinetic study for HIV-1 clade B and C gp120 proteins and METH impact DRD-2. Astrocytes (1 × 10^6^ cells/ ml) were cultured for 24 h without treatment (control), HIV-1 clade B gp120, HIV-1 clade C gp120 (0–100 ng/ ml) and METH (0–20 μM). Total cellular protein lysates were resolved by 4–15% SDS-PAGE run under the same experimental conditions and protein expression was analyzed by Western blots specific for clade B and C gp120 in DRD-2 (**A**) and METH effect DRD-2 (**B**). Fig. 3C and D show the % of band density. Band density data are expressed as the mean ± SE from three independent experiments.

The data presented in Fig. 3 illustrate the dose-dependent effect of HIV-1 clade B and C gp120 (A) and METH (B) on DRD-2 protein levels. These results demonstrate that clade B gp120 and METH significantly reduces DRD-2 protein levels compared to the untreated control whereas clade C gp120 had no effect on DRD-2. The densitometry analysis is presented in Fig. 3C,D respectively and the statistical analyses were performed by one-ANOVA with post-hoc test and one –way ANOVA method.

### Effect of intracellular expression and protein modification in DRD-2, CaMKs and CREBP by clade B and C gp120 with METH

**Figure 4:**
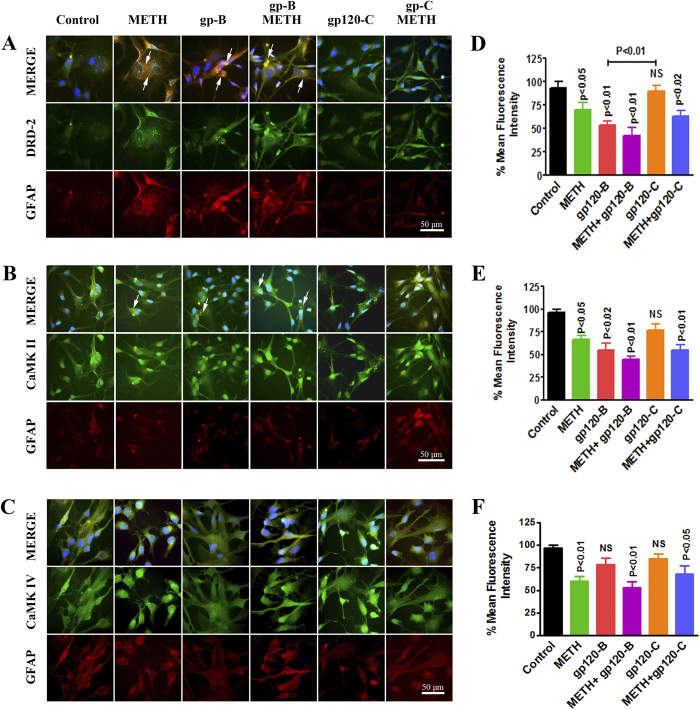
Intracellular expression of DRD-2, phospho-CaMK II, and phospho-CaMK IV. Astrocytes (1 × 10^6^ cells/ml) were cultured for 24 h without treatment or with METH (10 μM), clade B gp120 (50 ng/ ml), or clade C gp120 (50 ng/ ml) alone or METH in combination with clade B or C gp120. After incubation, the cells were fixed, permeabilized, and stained for (**A**) DRD-2, (**B**) phospho-CaMK II (**C**) phospho-CaMK IV (green) or GFPA (red) and DAPI (blue). The cells were imaged using a confocal microscope at 630x and analyzed using immunocytochemistry. Representative images from three individual experiments are shown. Figure 5D–F show the % of bars represent mean ± SE from three independent experiments.

**Figure 5:**
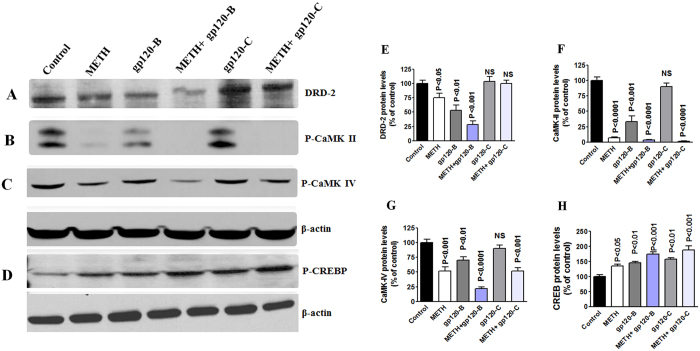
Effect of METH, gp120 clade B and clade C on CREBP. Astrocytes (1 × 10^6^ cells/ ml) were cultured for 24 h treatment with either METH (10 μM) alone or combined treatment with clade B gp120 (50 ng/ ml), or clade C gp120 (50 ng/ ml). Total cellular protein lysates were resolved by 4–15% SDS-PAGE run under the same experimental conditions and Protein expression was analyzed by Western blots for DRD-2 (**A**), CaMK II (**B**), CaMK IV (**C**) and CREBP (**D**). The densitometry analysis of DRD-2, (**E**) CaMK II (**F**), CaMK IV (**G**) and CREBP (**H**) show the % of band density. Band density data are expressed as the mean ± SE from three independent experiments.

We have used fluorescence microscopy to confirm the intracellular expression levels by DRD-2, CaMK II and CaMK IV in astrocytes. We determined the effect of HIV-1 clade B and C gp120 in the presence and absence of METH, which potentiates HIV-1-induced effects on the dopaminergic system and CaMKs expression in astrocytes. The data presented in Fig. 4 demonstrate that HIV-1 clade B and C gp120 induced a significant decrease in intracellular expression of DRD-2 (A), CaMK II (B) and CaMK IV (C) levels at 24 h, compared to the untreated control. Figures 4D–F are showing the % of mean fluorescence intensity by DRD-2, CaMK II and CaMK IV, respectively and the statistical analyses was performed using t-test with independent samples. These results demonstrate that METH and clade B gp120 significantly reduce DRD-2, phospho-CaMK II, phospho CaMK IV levels. However, HIV-1 clade C gp120 alone had no effect on DRD-2, phospho CaMK II and phospho-CaMK IV, but METH in combination with clade C gp120 significantly reduced phospho-CaMK, II and IV levels without affecting DRD-2. Furthermore, confirmatory studies were carried out by western blots to identify the modification of protein expression in dopaminergic system and CaMKs and induction of CREB using METH combined with either clade B gp120 or clade C gp120. Figure 5 shows the results on DRD-2 (A), CaMK II (B), CaMK IV(C) and CREB (D). These protein results confirm that the METH and clade B gp120 significantly reduce DRD-2, phospho-CaMK II, phospho CaMK IV levels. Interestingly, clade C gp120 combined with METH showed loss of p-CaMK II and stimulated p-CREB without affecting DRD-2 and p-CaMK IV. The densitometry analysis is presented in Fig. 5 DRD-2(E), CaMK II (F), CaMK IV (G) and CREBP (H) respectively.

### Effects of DRD-2 agonists and antagonists and CaMKs inhibitors

**Figure 6:**
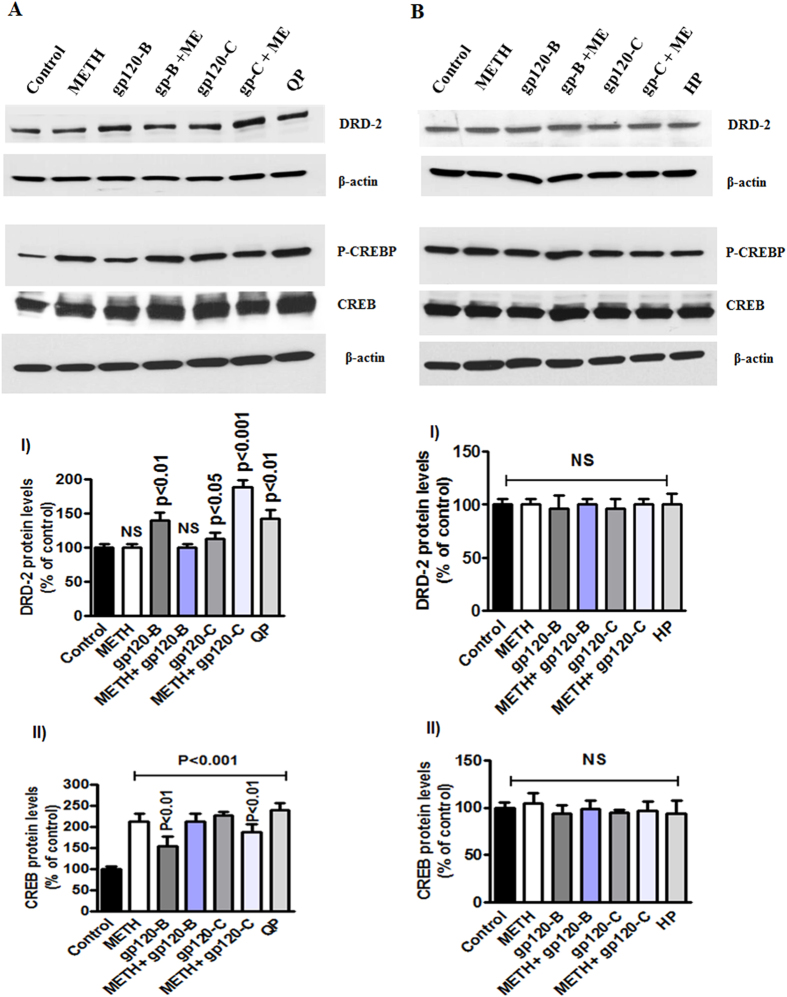
Protective and enhanced effects of DRD-2 agonists and antagonists. Astrocytes (1 × 10^6^ cells/ ml) were cultured for 24 h without treatment (control) or with METH, HIV-1 clade B gp120, or HIV clade C gp120 alone and 30 minutes pretreatment with the agonist quinpirole hydrochloride (QP)(10 μM) (**A**) or the antagonist haloperidol (HP) (10 μM) (**B**). Total cellular protein lysates were resolved by 4–15% SDS-PAGE run under the same experimental conditions and protein levels of DRD-2 and phospho-CREB were analyzed by Western blots. The densitometry analysis of DRD-2 and phospho-CREB show the % of band density respectively. Data are presented for three independent experiments.

**Figure 7:**
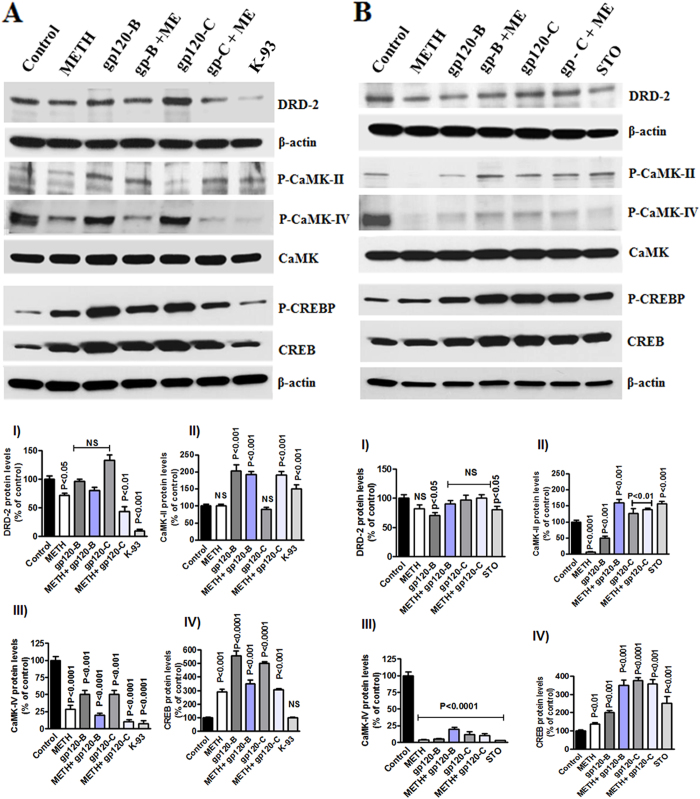
Effect of CaMKs inhibitors STO-609 and KN-93 in METH interact with HIV-1 clade B and C gp120. Astrocytes (1 × 10^6^ cells/ ml) were cultured for 24 h without treatment or with METH, HIV-1 clade B gp120, or HIV clade C gp120 alone and 30 minutes pretreatment with the CaMKK inhibitor STO-609 (**A**) or the CaMK II specific inhibitor KN-93 (10 μM) (**B**). Total cellular protein lysates were resolved by 4–15% SDS-PAGE run under the same experimental conditions and protein levels of DRD-2, p-CaMK II, p-CaMK IV and CREB were analyzed by Western blots. The densitometry analysis of DRD-2, p-CaMK II, p-CaMK IV and p-CREB show the % of band density respectively. Data are presented for three independent experiments.

In addition, we used the dopamine receptor agonist quinpirole hydrochloride (QP) and the dopamine receptor antagonist haloperidol (HP) to test the clade B and clade C gp120 with METH. The data presented in Fig. 6 show the results of treatment with the dopamine agonist QP (A) and antagonist HP (B) 30 minutes prior to treatment with METH, clade B gp120, or clade C gp120 alone or METH in combination with clade B or C gp120 for 24 hrs. The results demonstrate that the agonist QP failed to reverse the effects of METH and gp120 on DRD-2 and phospho-CREB whereas the antagonist HP reversed these effects. In addition, we investigated the effects of pretreatment with 1, 8-Naphthoylene benzimidazole-3-carboxylic acid (STO-609), a specific inhibitor of CaMKK, and KN-93, a CaMKII inhibitor, on astrocytes. The results demonstrate that (Fig. 7A) clade B gp120 and METH induce the dephosphorylation of CaMK II and CaMK IV and that this effect is maintained following co-administration of the CaMKs II inhibitor KN-93 (10 μM). Interestingly, the effects of METH alone or in combination with clade B or clade C on gp120 phospho-CaMK II and phospho-CaMK IV were not restored by KN-93. However, KN-93 significantly reduced phospho CREB in the nucleus. In contrast, the specific CaMKs inhibitor STO-609 had no effect on either CaMK II or CaMK IV in the cytoplasm (Fig. 7B). The observed effects of treatment with METH on phospho-CREB in nuclear extracts were reversed by STO-609. However, the combined effects of METH plus clade B or clade C gp120 were not reversed by STO-609. These data suggest that changes in gene expression levels correlate with changes in protein levels. However, inhibitor studies revealed that HIV subtypes B and C differentially regulate DRD-2 and CaMKs protein expression and CREB transcription, leading to distinct functional effects on neuropathogenesis in the CNS.

### Sequence analysis and structural model of HIV-1 clade B and C gp120

Sequence alignment and model of clade B and clade C gp120 is shown in Fig. 8. The important co-receptor binding sites such as V1, V2, V3, V4, α2 and N-glycosylation are highlighted. Figure 8A shows that clade B has 77% sequence similarity with clade C. The clade B and clade C have 17 conserved glycosylation sites (shown in blue color, whereas other non-conserved glycosylation sites are marked in red). Sequence alignment of clade B and clade C gp120 shows that the V1 region is highly variable with more gaps and that clade B is longer than clade C in V1 region. V4 region is slightly conserved, whereby clade B has five residues longer than clade C. The V2 and α2 regions are partially conserved; in contrast, V3 region is highly conserved. This suggested that V1 region is very distinct for each clade (Fig. 8B,C).

## Discussion

During the last decade, an intertwined epidemic of drug abuse and HIV-1 infection has emerged. Illicit drug abuse, including METH abuse, is a significant risk factor for HIV infection and AIDS disease progression^36^,^37^. METH is currently used in epidemic proportions worldwide and particularly in the U.S. The 2012 report reveals that 440,000 Americans age 12 or older are METH users^38^. Previous studies suggest that METH use and HIV-1 infection are independently associated with immune dysfunction^39^, which leads to neural impairments, such as neurotransmitter and dopaminergic dysfunctions^40^. The dopamine receptors and transporter play central roles in immune and CNS neurotransmission and are associated with neuro-pathological disorders implicated in HAND^41^,^42^. During HIV infection^22^,^43^, there is a reduction of dopamine expression and a disruption of dopaminergic function^44^,^45^, which is mediated by the dopamine transporter in the CNS^46^,^47^ and exacerbated by METH^48^. Dysfunction of the dopaminergic system, affecting DRD-2, DAT and intracellular Ca^2+^ levels, are found in the cerebrospinal fluid (CSF) of HIV associated dementia (HAD) patients^8^,^49^,^50^.

Studies have also demonstrated that the brains of METH users have a dramatically altered dopaminergic system^33^,^51^. Recently, Wang *et al*. (2004) reported that patients suffering from HAD exhibited a 13–20% reduction in dopamine transporter density compared to seronegative controls^8^. Further, the intracellular Ca^2+^ signal regulates multiple CaMKs signaling pathways in the CNS. Studies have demonstrated that CaMKs play a crucial role in ATP synthesis and inflammatory cytokine production during drug abuse and in HIV-infected subjects. The CaMKs signaling cascade affects cAMP enzyme activities and CREB transcription, which may lead to neuronal plasticity^49^. Acute and chronic administration of METH also alters CREB and leads to neuronal dysfunction^35^.

In addition, HIV-1 geographical variants and genetic polymorphisms may lead to a differential expression of HAD and HAND. HIV-1 clade B infections occur predominantly in North America, Western Europe, and Australia. In contrast, HIV-1 clade C is found in Africa, Latin America, and Asia^27^. Studies have demonstrated that HAND appears to be most common with HIV-1 clade B infections, whereas HAND occurs less frequently with HIV-1 clade C infection. This finding suggests that the prevalence of HAND may be correlated with different subtypes of HIV-1. However, toxic HIV-1 gp120 proteins have been found in high concentrations in the basal ganglia of patients with AIDS-dementia complex (ADC), and it is likely that these proteins play a significant role in the destruction of neurons that are involved in dopamine signal transmission. Previous studies demonstrated that HIV-1 clade B infection and clade B Tat protein induce more neuronal dysfunction than HIV-1 clade C infection and clade C Tat protein^52^,^53^,^54^. We have earlier reported that clade B and clade C, Tat and gp120 differentially impact immune and neuronal dysfunctions^55^,^56^,^57^. Here, we investigated whether HIV-1 B and C-derived gp120 differentially affects the dopaminergic system in human primary astrocytes. The main aim of this study is to analyze whether METH co-morbidity mimics similar or distinct functional effects when combined with HIV-1 clade B and C. However, there are currently no reports concerning the impact of clade B and C gp120 alone or in combination with METH on dopaminergic dysfunction and CaMKs dephosphorylation leading to CREB mediated neuronal toxicity.

In this study, we demonstrated for the first time that clade B gp120 decreased DRD-2 mRNA expression in astrocytes compared to clade C gp120 (Fig. 1A,B). In METH treated astrocytes DRD-2 and DAT expression are significantly down regulated (Fig. 1C,D). In addition, we found that the inhibition of DRD-2, DAT, CaMK II and CaMK IV gene and intracellular expression by clade B gp120 was potentiated by METH (Figs. 2,3 and 4). The expression of DRD-2, DAT, CaMK II and CaMK IV were not affected by clade C gp120; however, when clade C gp120 was combined with METH, these genes, proteins and intracellular expression of DRD-2 and CaMKs were significantly down regulated. Interestingly, CREB stimulation by METH together with clade B gp120 and clade C gp120 may affects both DNA binding and transactivation. Clade C gp120 combined with METH showed loss of CaMK II without affecting DRD-2 and CaMK IV, however, CREB was stimulated (Fig. 5). These studies suggest that only clade B actively impact the dopaminergic system whereas combined treatment with METH synergistically potentiates the dopaminergic system in both clades. Altogether, our results indicate that the effects induced by clade B gp120 are more potent than those induced by clade C gp120.

Intracellular Ca^2+^ levels play a pivotal role in dopaminergic function and subsequently affect CaMKs in METH users, leading to neuronal plasticity. Studies have demonstrated that increased intracellular Ca^2+^ levels activate ion channels and signaling intermediates of CaMK in HIV-infected patients^58^. Therefore, in this study, we examined whether HIV-1 gp120 from different clades and METH inhibit similar or distinct mechanisms of CaMKs signaling (Figs. 4,5). The results indicate that HIV-1 gp120 downregulates the dopaminergic system and that METH potentiate this effect. These studies suggest that patients infected with HIV-1B may have reduced DAT levels and increased levels of intra cellular Ca^2+^ compared to HIV-1C infected subjects. These results are consistent with earlier reports that the incidence of HAND is increased in patients with HIV-1 clade B neuropathogenesis^54^,^55^,^56^. These studies further confirm that the downregulation of CaMKs isoforms II and IV, and CREBP in HIV-1 clade B gp120-treated cells may lead to depression and cognitive disorders in HIV-infected patients and HIV-infected METH users.

The main observation in this report is that clade B and C gp120 differentially inhibit the dopaminergic systems and that METH potentiates these effects. However, pretreatment of astrocytes with agonist (QP) accelerates these effects (Fig. 6A) whereas antagonist (HP) protects gp120 induced DRD-2 and p-CREB effects (Fig. 6B). In these studies, clade B and C gp120 and/or METH differentially impact DRD-2 whereas CREBP is not affected by both clades. Interestingly, the inhibitors STO-609 and K-93 (Fig. 7A,B) differentially impact the dopaminergic pathway. Specifically, HIV clade C activates CREB phosphorylation in the absence of DRD-2 and CaMKs inhibition. This suggests that these two HIV-1 clades may have different signaling mechanisms and distinct functional effects. Studies have also reported that METH use and HIV infection affect the dopaminergic system, which implies that CaMKs-mediated changes in CREBP transcription lead to neuronal impairments^44^,^59^,^60^. In addition, studies using clade B and C gp120 in combination with agonists, antagonists and inhibitors revealed that these two HIV clades cause distinct functional effects via distinct signaling mechanisms. Overall, these mechanisms may underlie the observed differential expression of disease progression and neuronal impairments in patients infected with HIV clade B and C.

In this regard, studies have demonstrated that the sequence of the clade B envelope protein gp120 differs from that of clade C in the V3 and C3 regions, which play important roles in the function and structure of the protein^61^,^62^. In addition, the regional density of gp120 protein, as characterized by the number of charged residues in α2, is higher in clade B than in clade C. Recently, Gnanakaran *et al*. (2007) reported that the V4 loop shows extensive clade-dependent variation in length, with a shorter length in clade C than clade B^29^,^31^.

The genomic sequences and structural variation in the global subtypes, especially N-glycosulated proteins, have a wide role in neuropathogenic mechanisms in HIV infection. These distinct glycosulated proteins activate immune dysfunction in clade variations. Recent studies have shown HIV infection binding to CXCR4 and CXCL2 receptors, with clade binding variations depending on glycosylated proteins^63^,^64^. Therefore, we have analyzed the N-glycosylated structural sequence comparing clades B and C gp120 in V1-V4 loop region. The sequence alignment of clade B Bal and clade C-CN54 were shown in Fig. 8A. The co-receptor binding sites such as V1-V4, α2 and N-glycosylation sites are different in clade C -CN54 compared to clade-B Bal. Therefore, it is important to understand how these variable domains might influence the overall conformation of the native glycoprotein in the context of the functional effects caused by METH and HIV infection.

For further analysis, three dimensional structural models were developed using web based fully automated program SWISS-MODEL^65^, which yields two highly reliable models for both clade B and clade C structures, based on the template structures of PDB ID 2B4C and 3JWD. The first model was built from the gp120 core protein containing the V3 region of PDB ID: 2B4C^66^, which has 75% and 65% sequence identity with clade B (residue range 83–486) and clade C (residue range 83-471) respectively, but it lacks the V1 and V2 regions. V1/V2 gp120 regions are important for antibody binding but are not resolved in crystal structures. The second model was built from the recent structure (PDB ID: 3JWD) of gp120 with gp41 interactive region^67^. Model 2 has 70% and 64% of sequence identity with clade B and clade C respectively. It also includes both V1 and V2 regions, based on the sequence similarity using different structures, which are not part of the gp120. In Fig. 8B,C, the V1 and V2 regions adopt different secondary structural conformations for clade B and clade C, suggesting that this region is highly flexible and may form different conformations based on the antibody binding. The V3, V4 and α2 regions form similar secondary crystal structures of different gp120 proteins, such as clade B and C with V1, V2 and V3 regions may yield better understanding of the functions of these proteins. It is important to note that V3 region may adopt flexible orientation based on the different antibody bindings. Further structural analysis on the co-receptor binding sites such as V1 and V2 regions will be necessary to understand the binding mechanism of clade B and clade C and also how the clade variations affect differential neuropathogenic mechanisms leading to HAND.

In conclusion, HIV-1 gp120 subtypes differentially impact the dopaminergic system and regulate CREB transcription in cultured primary human astrocytes. It is very well established that METH, a potent central nervous stimulant with high abuse potential as well as human immunodeficiency virus (HIV)-1 are implicated in the progression of neurocognitive malfunction. Both have been shown to induce common neurodegenerative effects such as astrogliosis, compromised blood brain barrier (BBB) integrity, and excitotoxicity in the brain. However, the cellular and molecular mechanisms of astrocyte-mediated excitotoxicity in the context of METH and HIV-1 are undefined. Accordingly, in this study, it was observed that the distinct structure and sequence variation of clade B gp120 differentially impact DRD-2, DAT, CaMK II and CaMK IV mRNA, protein and intracellular expression compared to clade C gp120. Also, CREB stimulation is upregulated by both B and C gp120 and further METH co-treatment potentiated these effects hastening the process of neuropathogenesis. Also, recent evidence suggests astrocyte and neuron crosstalk mechanisms are involved in glutamate regulation and astrocytes play an essential role in the neuropathogenesis associated with METH/HIV-1-induced excitotoxicity and it is likely that similar mechanisms may be operative.

## Materials and Methods

### Reagents

Cell culture reagents were purchased from Sciencell (Carlsbad, CA, USA). The dopamine receptor-2 (DRD-2) and phospho-CaMK IV (The 196) antibodies were purchased from Santa Cruz Biotechnology (Santa Cruz, CA, USA). The mouse anti-CaMK IV antibody was purchased from BD Transduction Laboratories (San Jose, CA, USA). The phospho-CaMK II (The 196) antibodies were purchased from Signalway Antibody (Pearland, TX, USA). The anti-phospho CaMK II (The 196) antibodies were purchased from Phospho Solutions (Aurora, CO, USA). The rabbit polyclonal CREB antibodies where purchased from Abcam (Cambridge, MA, USA). The goat anti-rabbit IgG and goat anti-mouse IgG antibodies were purchased from Santa Cruz Biotechnology. Electrophoresis reagents were purchased from Bio-Rad (Richmond, CA, USA), and nitrocellulose membranes were purchased from Amersham Scientific, Piscataway, NJ. All other reagents were purchased from Sigma–Aldrich (St. Louis, MO, USA).

### HIV-1 clade-specific gp120 recombinant proteins

The HIV-1 clade B gp120 (Bal) and clade C gp120 (CN54) proteins were obtained from the NIH AIDS Research and Reference Reagent Program. The recombinant clade B and clade C gp120 proteins have >95% and >90% purity, respectively.

### Primary human astrocyte cultures

In this study, we used primary human astrocytes (HA), obtained from Sciencell (Cat# 1800), (Carlsbad, CA). The primary human astrocyte were grown in T-75 flasks containing basal medium (Cat#1801) with 10% fetal bovine serum, 50 units/ml of penicillin, astrocyte growth supplement, and 100 μg/ml of streptomycin. The cells were maintained in a humidified, 95% air and 5% CO2 atmosphere incubator at 37 °C. Cells, growth supplement and medium were obtained from Sciencell (Carlsbad, CA).

### RNA extraction and real-time quantitative PCR (Q-PCR)

Total RNA from astrocytes was extracted using a Qiagen kit (Invitrogen Life Technologies, Carlsbad, CA, USA) following the manufacturer’s instructions. The resulting total RNA (3 μg) was used to synthesize the first strand of cDNA. cDNA amplification was performed using primers specific for DRD-2 (Assay ID Hs 00241436), DAT (Assay ID Hs00997371), CaMK II (Assay ID, Hs 00392405); CaMK IV (Assay ID Hs 00241436), CREB (Assay ID, Hs 00392405) and β-actin (Assay ID, Hs99999903) (Applied Biosystems, Foster City, CA). β-actin was used as a housekeeping gene for normalizing the real-time PCR results. The relative abundance of each mRNA species was assessed using the brilliant Q-PCR master mix from Applied Biosystems and the Stratagene Mx3000P instrument, which detects and plots the increase in fluorescence versus the PCR cycle number to produce a continuous measure of PCR amplification. Relative expression was quantitated for each mRNA species, and the mean fold change in the expression of the target gene was calculated using the comparative CT method (Transcript Accumulation Index, TAI = 2^_ΔΔCT^). All data were controlled for the quantity of RNA input by performing measurements on the endogenous reference gene β-actin. In addition, RNA results from treated samples were normalized to results obtained using RNA from the control, untreated sample^68^.

### Western blot analysis

To assess modifications of DRD-2, and CREBP in astrocytes by HIV-1 clade B and C gp120 alone or in combination with METH, the cells were lysed using lysis buffer (Pierce, Rockford, IL) with 1x complete protease inhibitor cocktail. Equal amounts of total cellular proteins were resolved using 4–15% gradient polyacrylamide gel electrophoresis, transferred to a nitrocellulose membrane and incubated with primary antibodies. Immunoreactive bands were visualized using a chemiluminescence western blotting system according to the manufacturers’ instructions (Amersham).

### Analysis of DRD-2 and intracellular CaMKs expression

HIV-1 clade B and C gp120-induced intracellular DRD-2 and CaMKs (CaMK II and CaMK IV) expression was analyzed using fluorescent microscopy. Briefly, astrocytes (5 × 10^5^) were seeded and separately treated with the HIV-1 B and C gp120 proteins (50 ng/ ml) for 24 hrs. At the end of the incubation period, the cells were washed twice with PBS. The cells were fixed for 15 min in 4% paraformaldehyde in PBS at 4 °C. The cells were permeabilized with 5% BSA, 0.1% Triton X-100 for 15 min at 4 °C and then incubated overnight at 4 °C with antibodies against the astrocyte marker GFAP, DRD-2, phospho-CaMK II and phospho-CaMK IV. The slides were washed in PBS and incubated with FITC-conjugated goat anti-rabbit IgG (Santacruz) and PE-conjugated goat IgG secondary antibodies (Jackson Immuno Research Laboratories, West Grove, PA, USA), followed by Hoechst 33258 (10  μg/mL) for 10 minutes. The slides were then washed with PBS, and intracellular DRD-2, p-CaMK II and p-CaMK IV fluorescence was examined microscopically.

### Statistical analysis

Data were analyzed using GraphPad Prism software. Comparisons between groups were performed using either t-test with independent samples or one-way ANOVA post hoc t-test. Differences were considered significant at p ≤ 0.05. Data are expressed as mean ± SE. Experiments were performed at least three times in triplicates.

## Additional Information

**How to cite this article**: Samikkannu, T. *et al*. HIV Subtypes B and C gp120 and Methamphetamine Interaction: Dopaminergic System Implicates Differential Neuronal Toxicity. *Sci. Rep*. **5**, 11130; doi: 10.1038/srep11130 (2015).

## Figures and Tables

**Figure 8:**